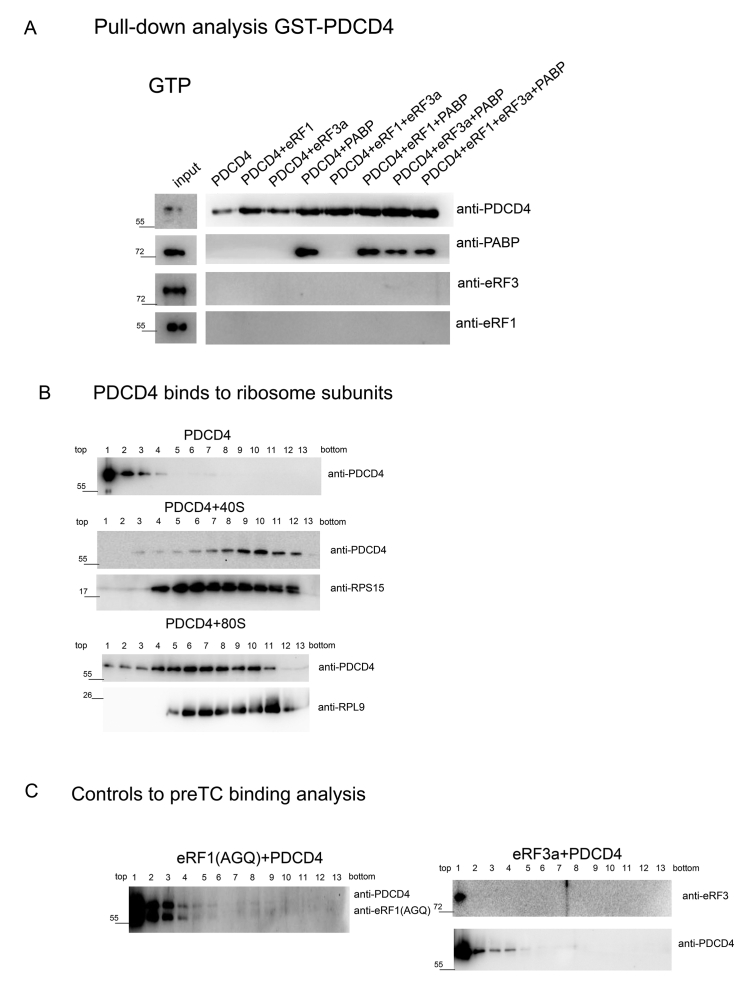# Discovery of a novel role of tumor suppressor PDCD4 in stimulation of translation termination

**DOI:** 10.1016/j.jbc.2021.101269

**Published:** 2021-10-01

**Authors:** Ekaterina Shuvalova, Tatiana Egorova, Alexander Ivanov, Alexey Shuvalov, Nikita Biziaev, Sabina Mukba, Nikolay Pustogarov, Ilya Terenin, Elena Alkalaeva

**Affiliations:** 1 Engelhardt Institute of Molecular Biology, The Russian Academy of Sciences, Moscow, Russia; 2 Center for Precision Genome Editing and Genetic Technologies for Biomedicine, Moscow, Russia; 3 A.N. Belozersky Institute of Physico-Chemical Biology, Lomonosov Moscow State University, Moscow, Russia

**Keywords:** translation termination, ribosome, PDCD4, eRF1, eRF3, eIF4A, eukaryotic initiation factor 4A, eRF1, eukaryotic release factor 1, eRF3a/c, eukaryotic release factor 3a/c, PABP, poly (A)-binding protein, PDCD4, programmed cell death protein, preTC, pretermination complex, postTC, posttermination complex, RPL, ribosomal protein L, RPS, ribosomal protein S, TC, termination complex

## Abstract

Programmed cell death 4 protein (PDCD4) regulates many vital cell processes, although is classified as a tumor suppressor because it inhibits neoplastic transformation and tumor growth. For example, PCDC4 has been implicated in the regulation of transcription and mRNA translation. PDCD4 is known to inhibit translation initiation by binding to eukaryotic initiation factor 4A and elongation of oncogenic c- and A-myb mRNAs. Additionally, PDCD4 has been shown to interact with poly(A)-binding protein (PABP), which affects translation termination, although the significance of this interaction is not fully understood. Considering the interaction between PABP and PDCD4, we hypothesized that PDCD4 may also be involved in translation termination. Using *in vitro* translation systems, we revealed that PDCD4 directly activates translation termination. PDCD4 stimulates peptidyl-tRNA hydrolysis induced by a complex of eukaryotic release factors, eRF1-eRF3. Moreover, in combination with the PABP, which also stimulates peptide release, PDCD4 activity in translation termination increases. PDCD4 regulates translation termination by facilitating the binding of release factors to the ribosome, increasing the GTPase activity of eRF3, and dissociating eRF3 from the posttermination complex. Using a toe-printing assay, we determined the first stage at which PDCD4 functions—binding of release factors to the A-site of the ribosome. However, preventing binding of eRF3 with PABP, PDCD4 suppresses subsequent rounds of translation termination. Based on these data, we assumed that human PDCD4 controls protein synthesis during translation termination. The described mechanism of the activity of PDCD4 in translation termination provides a new insight into its functioning during suppression of protein biosynthesis.

Programmed cell death 4 protein (PDCD4) is a broad regulator of vital processes occurring in cells, such as cell cycle progression (1), transcription (2, 3), and translation (4, 5). Additionally, PDCD4 is classified as a tumor suppressor (6). A decrease in PDCD4 expression can be accompanied by the development of malignant tumors, including the lung, colon, liver, and breast tumors, as well as glioblastomas (7, 8, 9, 10). This is because of increased motility and invasion of tumor cells (11) and suppression of DNA damage response pathways (12). One of the suppression mechanisms of tumor formation by PDCD4 occurs through the translation inhibition of a set of genes involved in proliferation, invasion, and metastasis of tumor cells (13, 14). Moreover, previous studies have reported on the proinflammatory role of PDCD4 (15), as well as the function of PDCD4 in the development of obesity and atherosclerosis (16, 17), where it participates in the formation of stress granules in response to oxidized low-density lipoproteins (16). An increase in PDCD4 expression at the mRNA and protein levels can occur in response to a variety of apoptosis inducers, thus affecting multiple signaling pathways (8, 18, 19), as well as in senescent cells (20). Accordingly, PDCD4 overexpression inhibits cancer cell proliferation and tumor growth (10).

The PDCD4 protein can migrate between the nucleus and cytoplasm as it contains two nuclear export signals. Under normal growth conditions, PDCD4 is mainly located in the nucleus; however, PDCD4 is predominantly cytoplasmic upon serum withdrawal (21). The localization of PDCD4 is regulated by Akt kinase; phosphorylation of S457 on the PDCD4 protein by Akt kinase tags it for nuclear translocation (22).

The mechanism of PDCD4 functioning in the cytoplasm appears to be closely related to the regulation of protein translation. PDCD4 contains the N-terminal RNA-binding domain and two MA3 domains located in the central and C-terminal regions of the protein (Fig. 1*A*). The MA3 domain belongs to the families of HEAT (Huntingtin, elongation factor 3, protein phosphatase 2A, and TOR1 kinase) repeat domains. HEAT is a structural motif consisting of repeats of two α-helices connected by a short loop (23). The MA3 domains are approximately 120 amino acids long and contain seven or eight α-helices, which are also found in other proteins, including eukaryotic translation initiation factor 4G (eIF4G) and nuclear cap-binding protein 80 (23). The MA3 domains of PDCD4 interact with eIF4A (Fig. 1*A*) to suppress its RNA helicase activity, thus suppressing the translation of mRNA with structured 5′UTRs (4). As shown by a yeast two-hybrid assay, the C-terminal region of PDCD4 weakly interacts with ribosomal protein L5 (RPL5) and strongly interacts with RPS13, which was confirmed by *in vitro* binding experiments (20). The N-terminus of PDCD4 contains an unstructured sequence that undergoes multiple phosphorylation events (22). PDCD4 interacts with the N-terminal domain of the coding region of the oncogenic mRNAs c- and A-myb to inhibit their translation at the elongation stage of translation (5, 24). Additionally, the N-terminus of PDCD4 interacts with poly (A)-binding protein (PABP), which is involved in maintaining mRNA stability, and implementing effective translation initiation and termination (25, 26). Only full-length PABP efficiently binds to the N-terminus of PDCD4 (Fig. 1*A*), and the major site of this interaction is with the RNA recognition motif 1 to 2 (RRM) domains of PABP.

**Figure 1:**
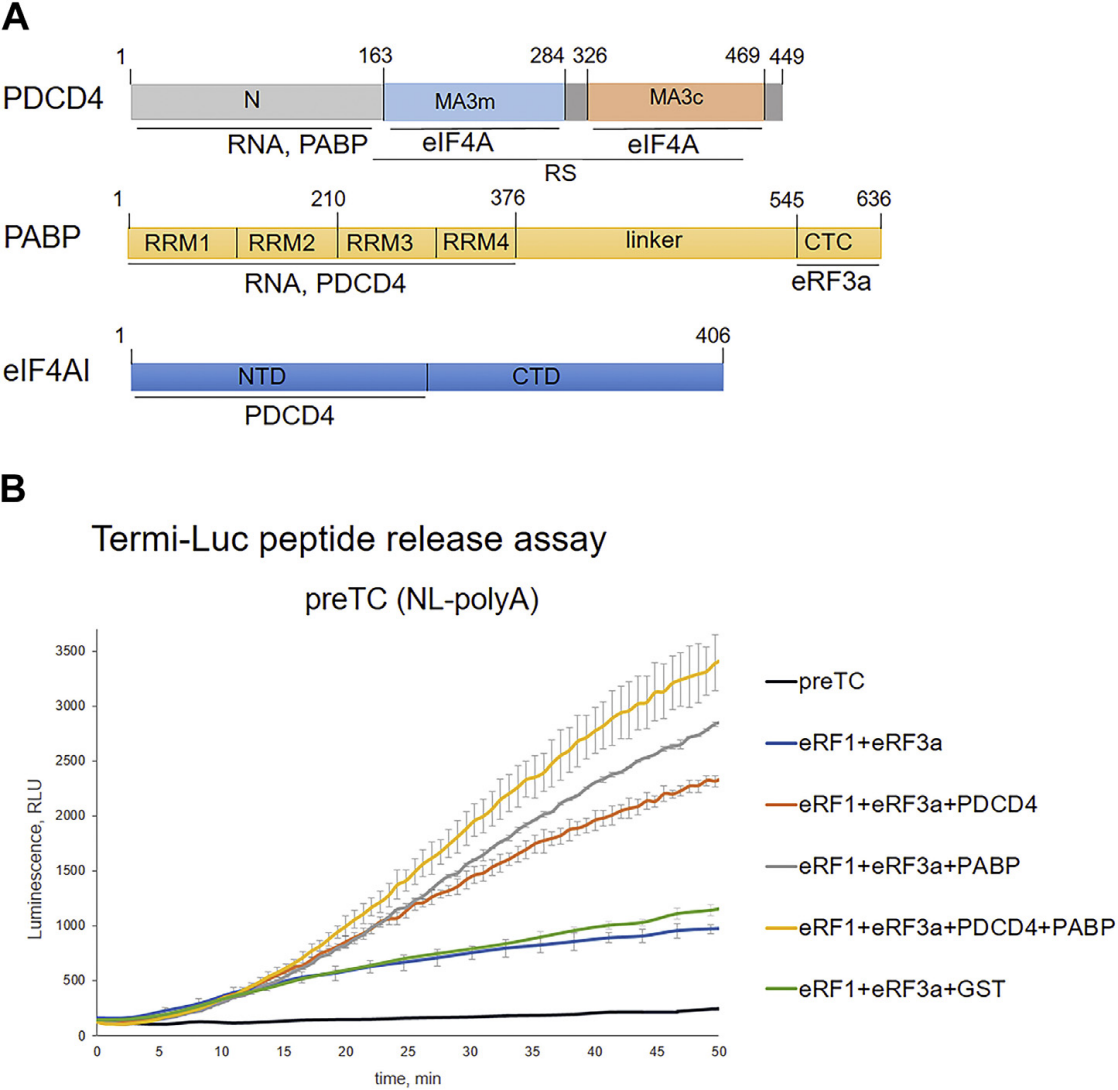
**PDCD4 enhances translation termination independently of PABP.***A*, interaction scheme of PDCD4, PABP, eIF4AI domains. *B*, Termi-Luc peptide release assay in the presence/absence of PDCD4, PABP, and the release factors. Time progress curves showing luminescence (in relative luminescence units, RLU) with NLuc luciferase released from ribosome complex upon treatment with the proteins of interest (n = 3). The error bars represent the standard mean deviation.

It has also been shown that PABP activates translation termination (26). Termination of eukaryotic translation occurs when one of the three stop codons (UAA, UGA, or UAG) of mRNA reaches the ribosomal A site. This process is catalyzed by two factors: eukaryotic release factor 1 (eRF1) and eRF3 (27). eRF1 contains N, M, and C domains and recognizes stop codons at the A site of the ribosome *via* its N domain (28, 29, 30). eRF3 is a translational GTPase that interacts with eRF1 (31). eRF3 consists of a highly variable unstructured N terminus, a GTPase G domain, and two C-terminal domains (2 and 3) (30, 32). Domain 3 of eRF3 interacts with the C domain of eRF1 and stimulates eRF1 activity through the hydrolysis of GTP (30, 33). The formation of the eRF1-eRF3 complex allows for eRF1 to adopt a certain conformation that provides effective hydrolysis of peptidyl-tRNA (29). PABP increases the binding efficiency of the human release factors to the ribosome through interactions with the N-terminal domain of eRF3a (26).

Considering the direct interaction of PABP and PDCD4, we hypothesized that PDCD4 may also be involved in translation termination, which has not previously been demonstrated. To investigate the possible effect of PDCD4 on the termination of eukaryotic translation, we used a reconstituted mammalian translation system (29) as well as pretermination complexes purified from rabbit reticulocyte lysate (RRL). We demonstrated that human PDCD4 binds to the pretermination and termination complexes (preTC and TC, respectively) and confirmed its involvement in translation termination. We show that PDCD4 stimulates translation termination and propose a model for its function.

## Results

### PDCD4 stimulates peptide release independently of PABP

Previously, we showed that PABP facilitates translation termination *via* interaction with eRF3а (26). Since PDCD4 also interacts with PABP, it may affect translation termination as well. To study the activity of PDCD4, the cDNA sequence encoding human PDCD4 was cloned into the pGEX-6p-1 vector and expressed in *Escherichia coli* BL21 cells. The resulting glutathione S-transferase (GST)-tagged PDCD4 or PDCD4 without the tag (after removal thereof by TEV protease) was used in the experiments.

**Supplemental Figure S1:**
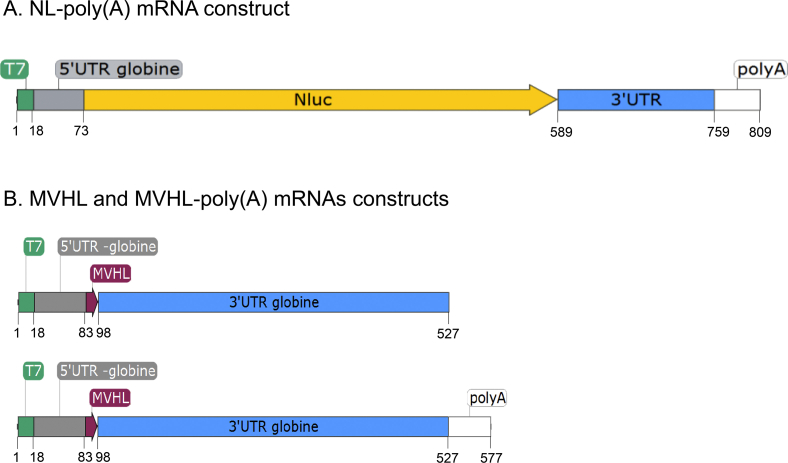


The final step of translation termination is the hydrolysis of peptidyl-tRNA and the release of the nascent peptide from the ribosome. Analysis of peptide release efficiency was performed using the highly sensitive Termi-Luc approach developed recently by Susorov *et al.* (34). PreTCs containing Nano luciferase (Fig. S1*A*) was fixed in the P-site by the mutant eRF1(AGQ), which is unable to hydrolyze peptidyl-tRNA to accomplish the process. These complexes were purified from the rabbit reticulocyte lysate (RRL) by sucrose density centrifugation at the high ionic strength, which resulted in eRF1(AGQ) dissociation from the ribosome. The addition of release factors to purified preTC(NL-polyA) triggered peptidyl-tRNA hydrolysis and the release of NLuc from the ribosome. This, in turn, can be detected by luminescence. To address the possible effect of PDCD4 in the complex with PABP on the translation termination process, eRF1, eRF3a, PDCD4, and PABP were added to the purified preTC(NL-polyA) simultaneously or in different combinations, and the luminescence was measured as a function of time.

Clearly, PDCD4 activates translation termination in the presence of PABP and, quite unexpectedly, in its absence (Fig. 1*B*). Yet, the proteins act synergistically, as the joint addition of PDCD4 and PABP increased peptide release more strongly than the separate addition of proteins. To ensure that the effect of PDCD4 is not associated with the stabilization of release factors or the nonspecific prevention of their sorption in test tubes because of very low eRF concentrations, we added GST to the reaction in the presence of release factors as a negative control. This addition revealed no effect on peptide release.

**Supplemental Figure S2:**
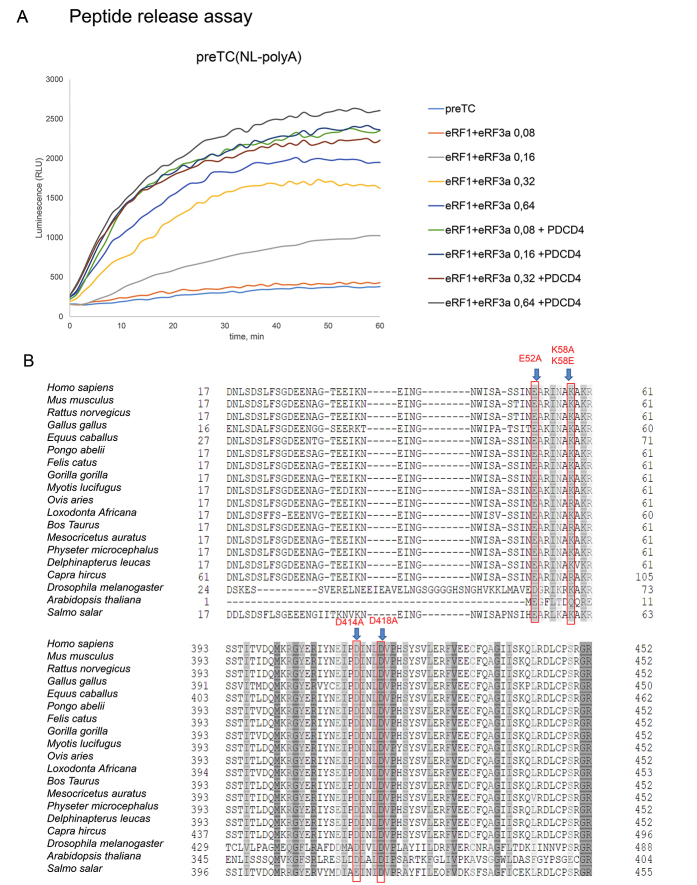


Importantly, we used a low concentration of the release factors, to detect the maximum stimulation of PDCD4. The titration curves of the release factors demonstrated that the efficiency of PDCD4 stimulation of peptide release depended on the concentration of release factors (Fig. S2*A*). Thus, the stimulation of peptide release from the terminating ribosome by PDCD4 indicates its involvement in the process of human translation termination.

The expected relative *in vivo* levels of PDCD4 and release factors were determined by the analysis of published mass spectrometry data (Table 1) (35, 36, 37). In most cell lines, the level of PDCD4 was lower than that of eRF1 and eRF3; however, in HEK 293, LnCap, and Jurkat cell lines, the level of PDCD4 was comparable to or even higher than that of eRF1. Therefore, the amount of protein used may reflect physiological conditions in some cell lines or specific conditions, such as cellular stress or apoptosis.

**Table 1:** Number of protein molecules per cell

Cell lines	eRF1	eRF3a	PDCD4	PABP
HeLa^a^	995,5	1930,1	837	2523,1
U2OS^b^	140,2	52,9	1,1	547,7
HEK 293^c^	5972,4	4956,4	2436,5	10353,9
HELA^c^	2763,7	2782,3	622,3	5086,1
GAMG^c^	3199,4	1438,7	477,1	6582,5
A549^c^	2794,7	1009,8	35,8	4512,3
HepG2^c^	2377,3	2212,1	95,6	2774,4
Jurkat^c^	931,9	911,4	1310,8	1967,7
K562^c^	1858,9	1211,2	96,5	2782,5
LnCap^c^	2370,1	1310,3	2886,8	5953,9
MCF7^c^	3860,2	4067,5	21,8	9794
RKO^c^	1686	1068,9	121	3072,9
U2OS^c^	4589,9	2997,5	268,9	10080,5

These data have been reorganized from mass spectrometry data reported previously.

iBAQ is the sum of all the peptide intensities divided by the number of observable peptides of a protein.

a Number per cell × 10^3^ (35).

b Number per cell × 10^3^ (36).

c iBAQ × 10^4^ (37).

### PDCD4 stimulates termination complex formation

**Figure 2:**
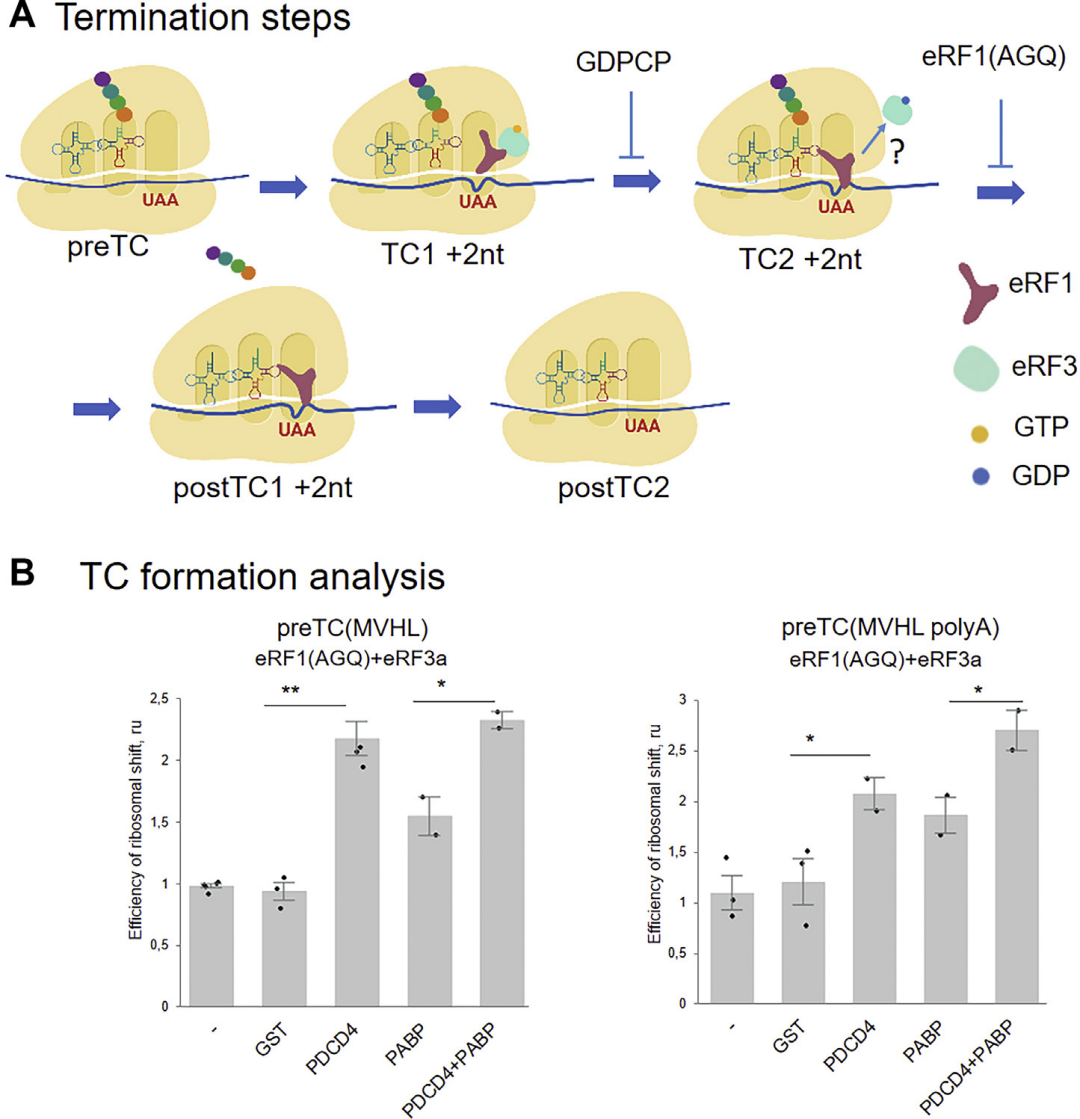
**Effect of PDCD4 on TC2formationin the presence/absence of PABP.***A*, translation termination steps. Addition of GDPCP to the translation system stabilizes ribosome position according to TC1 +2 nt, addition of the eRF1(AGQ) stabilizes ribosome position according to TC2 +2 nt. *B*, TC formation analysis according to efficiency of ribosomal shift during stop-codon recognition in the presence/absence of PDCD4 and PABP on MVHL mRNA or MVHL-polyA mRNA. The background was subtracted and each mean value was normalized to the value of eRF1(AGQ)+eRF3a. The *black dots* show the replicate values. The error bars represent the standard error. ∗∗*p* < 0.01, *t* test, ∗*p* < 0.05, *t* test.

In a complementary approach, we assayed a PDCD4 functioning in the reconstituted *in vitro* mammalian translation system (29). This system was assembled from the purified initiation and elongation translation factors, ribosomal subunits, tRNAs, and model MVHL-mRNA (Fig. S1*B*). As a result, a preTC(MVHL) containing a stop codon at the A site of the ribosome and peptidyl-tRNA at the P site was formed. After purification of the preTC(MVHL) from the unbound translation factors by sucrose gradient centrifugation, it was possible to study the influence of individual proteins on the subsequent translation termination stages. During translation termination, eRF1, in the complex with eRF3, recognized the stop codon and formed a termination complex 1 (TC1); then, eRF3 hydrolyzed GTP and positioned eRF1 in the peptidyl transferase center of the ribosome, forming TC2 (Fig. 2*A*). This triggered peptidyl-tRNA hydrolysis, and the complex passed into the posttermination stages (postTC1 and postTC2), followed by ribosome recycling. Addition of the non-hydrolysable GTP analogue, GDPCP, to the translation termination inhibited GTP hydrolysis and thus transition to the TC2 stage (Fig. 2*A*). The G183A mutant of eRF1 (eRF1(AGQ)), which was unable to hydrolyze peptidyl-tRNA, inhibited the transition to the postTC and freezed the termination process at the TC2 stage (Fig. 2*A*). Therefore, it is possible to investigate events occurring during TC formation in a great detail (38).

**Figure 3:**
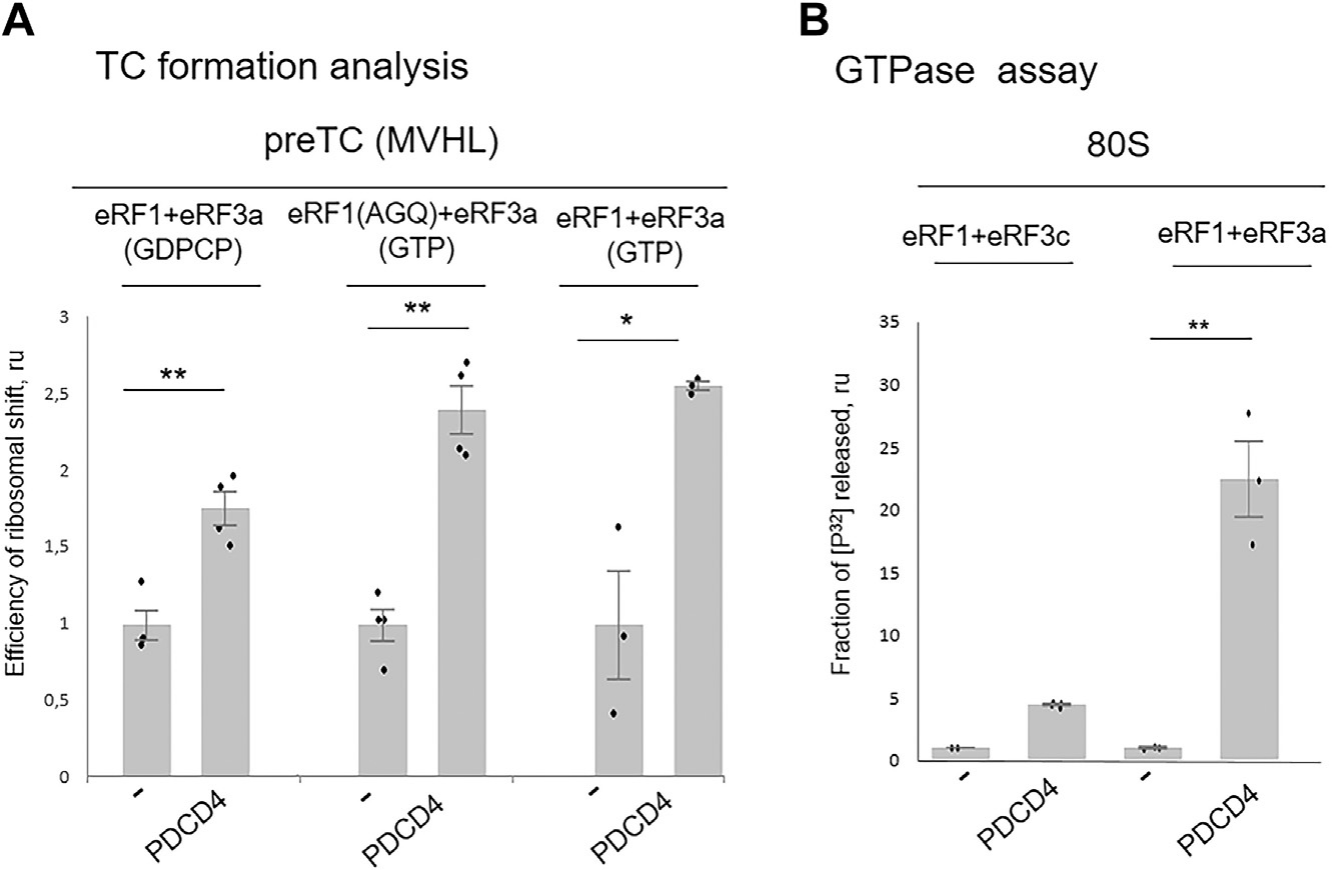
**Effect of PDCD4 on TC formation and GTPase activity of eRF3.***A*, TC1, TC2, and postTC formation analysis in the presence/absence of PDCD4 or GST as a negative control on MVHL mRNA. *B*, GTPase activity of eRF3a or eRF3c mixed with 80S and eRF1 in presence/absence of PDCD4. The background was subtracted and each mean value was normalized to the value of eRF1(AGQ)+eRF3 or eRF1+eRF3 activity. *Black dots* show the replicate values. The error bars represent the standard error, (n = 3) ∗∗*p* < 0.01, *t* test, ∗*p* < 0.05, *t* test.

**Supplemental Figure S3:**
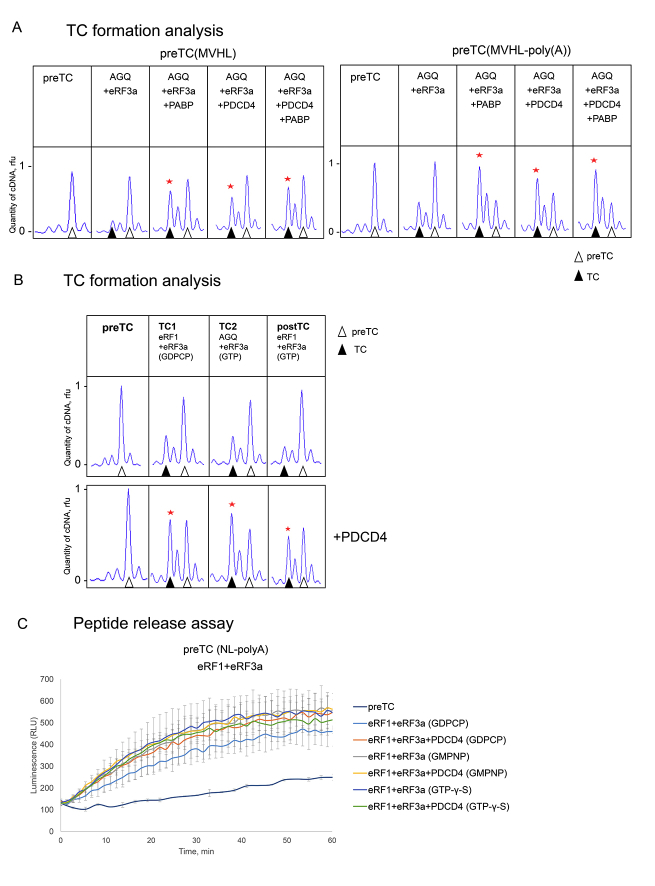


To study the effect of PDCD4 on the formation of termination complexes, PDCD4 along with eRF1 or eRF1(AGQ) and eRF3a was incubated with preTC(MVHL) in the presence of GTP or GDPCP, and the efficiency of TC1, TC2, and postTC1 formation was analyzed using a fluorescent toe-print assay (Figs. 2*B*, 3*A*, S3, *A* and *B,* and S4). This allowed for the detection of the position of stable ribosomal complexes on mRNA, using reverse transcription with a fluorescently labeled primer that anneals downstream of the putative ribosome-mRNA complex site. During stop codon recognition by eRF1, the ribosome undergoes a rearrangement, which manifests itself as a 1 to 2 nucleotide shift of the toe-print signal to the 3′ end (39). We revealed that the addition of PDCD4 increased the amount of all three tested ribosomal complexes, TC1, TC2, and postTC1 (Figs. 3*A* and S3*B*), indicating the involvement of PDCD4 in all stages of translation termination. It should be noted that we observed stimulation of TC1 formation in the presence of GDPCP by PDCD4, but it was weaker than in the presence of GTP.

To study the possible effect of PDCD4 in the complex with PABP on TC2 formation, PDCD4, eRF1(AGQ), eRF3a, and PABP were incubated with preTC(MVHL), and the efficiency of TC2 formation was analyzed. We showed that PABP did not interfere with PDCD4 in the stimulation of TC2 formation and even increased the efficiency of TC2 formation in the presence of a poly(A) tail (Figs. 2*B* and S3*A*).

Therefore, we confirmed PDCD4 activity in translation termination observed in peptide release experiments and demonstrated that this protein is involved in different termination steps.

### PDCD4 stimulates GTPase activity of eRF3

One of the intermediate stages of translation termination is GTP hydrolysis performed by eRF3, which occurs after eRF1-eRF3 binding to the stop codon in the ribosome. Using the GTPase test with [^32^P]-labeled GTP, we detected an increase in GTPase activity of the full-length eRF3a or its truncated form eRF3c, in the presence of PDCD4 (Fig. 3*B*). Interestingly, PDCD4 stimulated the GTPase activity of eRF3a more strongly than eRF3c. This finding correlates with the stimulation of translation termination in previously described experiments. To demonstrate the key role of PDCD4 in the GTPase activity of eRF3, we performed peptide release experiments with the non-hydrolyzable GTP analogues GDPCP, GMPPNP, and GTP-γ-S (Fig. S3*C*). When the GTP analogues were added to peptide release, no stimulation of translation termination by PDCD4 was detected. Thus, stimulation of peptide release by PDCD4 is performed *via* activation of the GTPase reaction on the ribosome.

### Minimum required TC components for activation by PDCD4

**Figure 4:**
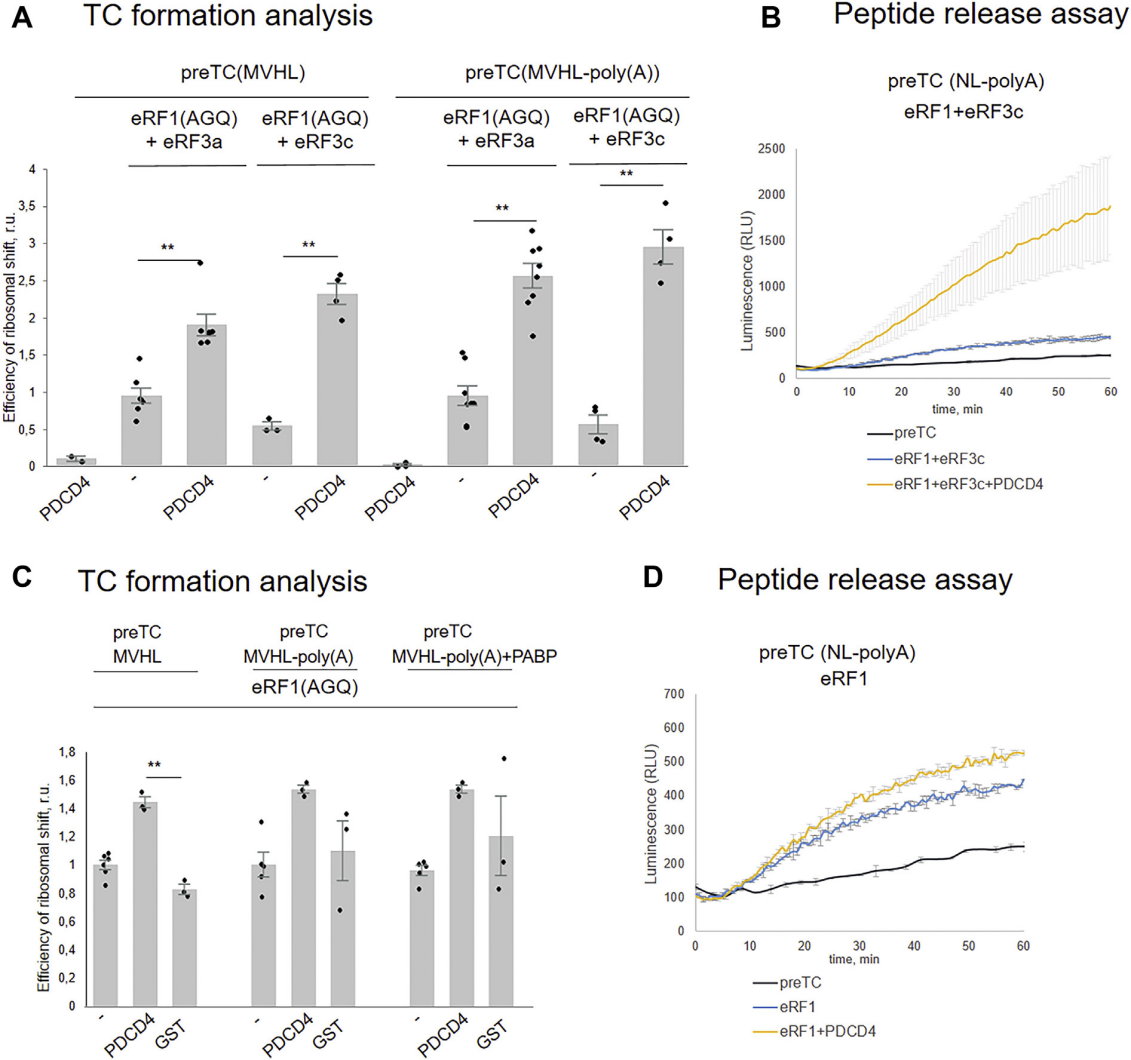
**Minimal required components of translation termination apparatus to be activated by PDCD4.***A*, TC formation analysis with eRF1 (AGQ)+eRF3a/eRF3c in the presence/absence of PDCD4 on MVHL mRNA and MVHL-poly(A). The background was subtracted and each mean value was normalized to the value of eRF1 (AGQ)+eRF3a/eRF3c. The *black dots* show the replicate values. The error bars represent the standard error, ∗∗*p* < 0.01, *t* test, ∗*p* < 0.05, *t* test. *B*, Termi-Luc peptide release assay showing level of peptidyl-tRNA hydrolysis induced by eRF1+eRF3c in the presence/absence of PDCD4. Time progress curves showing luminescence (in relative luminescence units, RLU) with NLuc luciferase released from ribosome complex upon treatment with the proteins of interest (n = 3). The error bars represent the standard mean deviation. *C*, TC formation analysis with eRF1 (AGQ) in the presence/absence of PDCD4 or GST as a negative control on MVHL mRNA, MVHL-poly(A) or MVHL-poly(A)+PABP. *D*, Termi-Luc peptide release assay showing level of peptidyl-tRNA hydrolysis induced by eRF1 in the presence/absence of PDCD4 (n = 3).

**Supplemental Figure S4:**
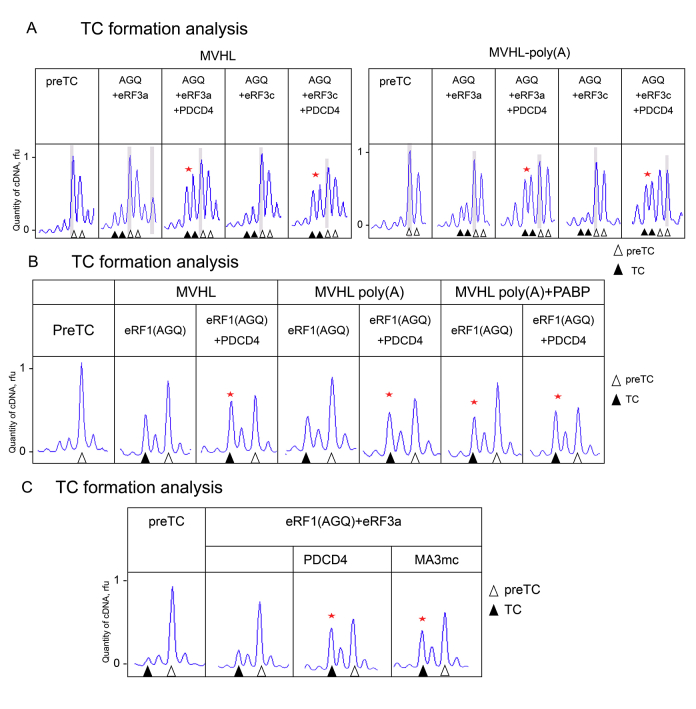


Previously, the activity of the N-terminal truncated eRF3a form, eRF3c, in translation termination was shown (40). It was also shown that eRF3c is unable to bind to PABP (32). We tested whether PDCD4 affects the termination induced by this protein. We revealed that PDCD4 stimulated the efficiency of TC formation in the presence of eRF3c and eRF1(AGQ) (Figs. 4*A* and S4*A*). Moreover, in contrast to GTPase activity, PDCD4 stimulated TC formation induced by eRF3c more strongly than that by eRF3a. The activity of both forms of eRF3 depended on the presence of a poly(A) tail because differences were found for the polyadenylated and deadenylated mRNAs. Therefore, the interaction of PDCD4 with the truncated form, eRF3c, is sufficient to stimulate translation termination. This result was further confirmed by the peptide release assay; the addition of PDCD4 stimulated the release of NLuc from the preTC when the termination was induced by eRF1 and eRF3c (Fig. 4*B*).

In the presence of eRF1 alone, PDCD4 slightly promoted TC formation independent of the poly(A) tail and PABP (Figs. 4*C* and S4*B*), but it was unable to stimulate the NLuc release from the preTC (Fig. 4*D*). Therefore, the minimum required release factor to be activated by PDCD4 is the eRF1-eRF3c complex.

**Figure 5:**
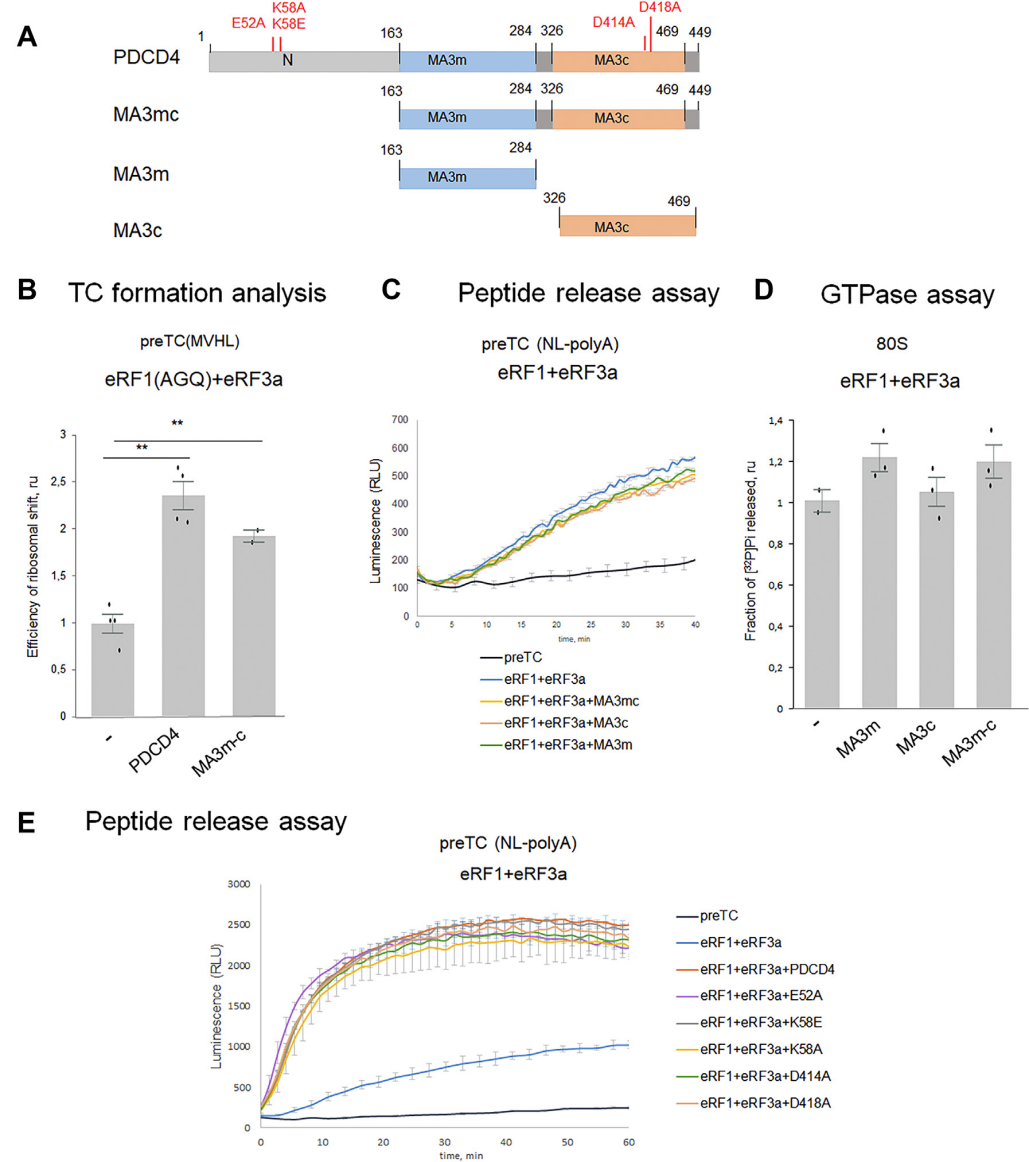
**Activity of domains and mutant forms of PDCD4 in translation termination.***A*, scheme of PDCD4 point mutants and domains. *B*, TC formation efficiency in presence of eRF1 (AGQ)+eRF3a and PDCD4 or MA3m-c domain of PDCD4. *C*, Termi-Luc peptide release assay in the presence of eRF1+eRF3a and MA3m, Ma3c, MA3m-c domains of PDCD4 (n = 3). *D*, GTPase assay in the presence of 80S subunits and eRF1+eRF3c with PDCD4 and MA3m, MA3c or MA3m-c domains. *E*, Termi-Luc peptide release assay in the presence of eRF1+eRF3a and PDCD4 wt or PDCD4 point mutants: E52A, K58E, K58A, D414A, and D418A (n = 2). For TC formation and GTPase analysis, the background was subtracted and each mean value was normalized to the value of eRF1 (AGQ)+eRF3a/eRF3c. The *black dots* show the replicate values. The error bars represent the standard error, ∗∗*p* < 0.01, *t* test, for peptide release analysis: time progress curves showing luminescence (in relative luminescence units, RLU) with NLuc luciferase released from ribosome complex upon treatment with the proteins of interest (n = 2). The error bars represent the standard mean deviation.

To elucidate the functions of individual PDCD4 domains, MA3m-c, MA3m, and MA3c (Fig. 5*A*), we determined their effects on TC formation and the release of NLuc. In the presence of both release factors eRF1(AGQ) and eRF3a, MA3m-c stimulated TC formation (Figs. 5*B* and S4*C*), but not the release of NLuc from the preTC (Fig. 5*C*). The MA3m and MA3c domains of PDCD4 were also unable to activate peptide release in the presence of eRF1-eRF3a. None of the truncated forms of PDCD4 was active in the GTPase test (Fig. 5*D*). Thus, the N domain of PDCD4 is required for functioning in translation termination. Therefore, the minimum set of components for translation termination in the presence of PDCD4 includes full-length eRF1, PDCD4, and truncated eRF3c.

### eIF4A does not affect activity of PDCD4 in termination

The best characterized binding partner of the PDCD4 is the translational helicase eIF4A. To elucidate if it affects the PDCD4 activity in translation termination, we first studied influence of point mutations that alter the PDCD4 interaction with eIF4A mutations (41, 42, 43). We expressed and purified five mutant variants of the protein—E52A, K58E, K58A, D414A, D418A (Figs. 5*A* and S2*B*). Mutants at positions 52 and 58 are located in the conserved positively charged N**E**ARINA**K**AKRRLRKN region of the N domain involved in PDCD4 binding to RNA (21). The conservative negatively charged residues, D414 and D418, are located in the MA3c domain and are involved in the binding of PDCD4 with initiation translation factor eIF4A (4). All mutants were tested using the peptide release assay (Fig. 5*E*), but none of the mutations affected the activity of PDCD4 in translation termination. Consequently, none of the tested residues were involved in translation termination.

**Figure 6:**
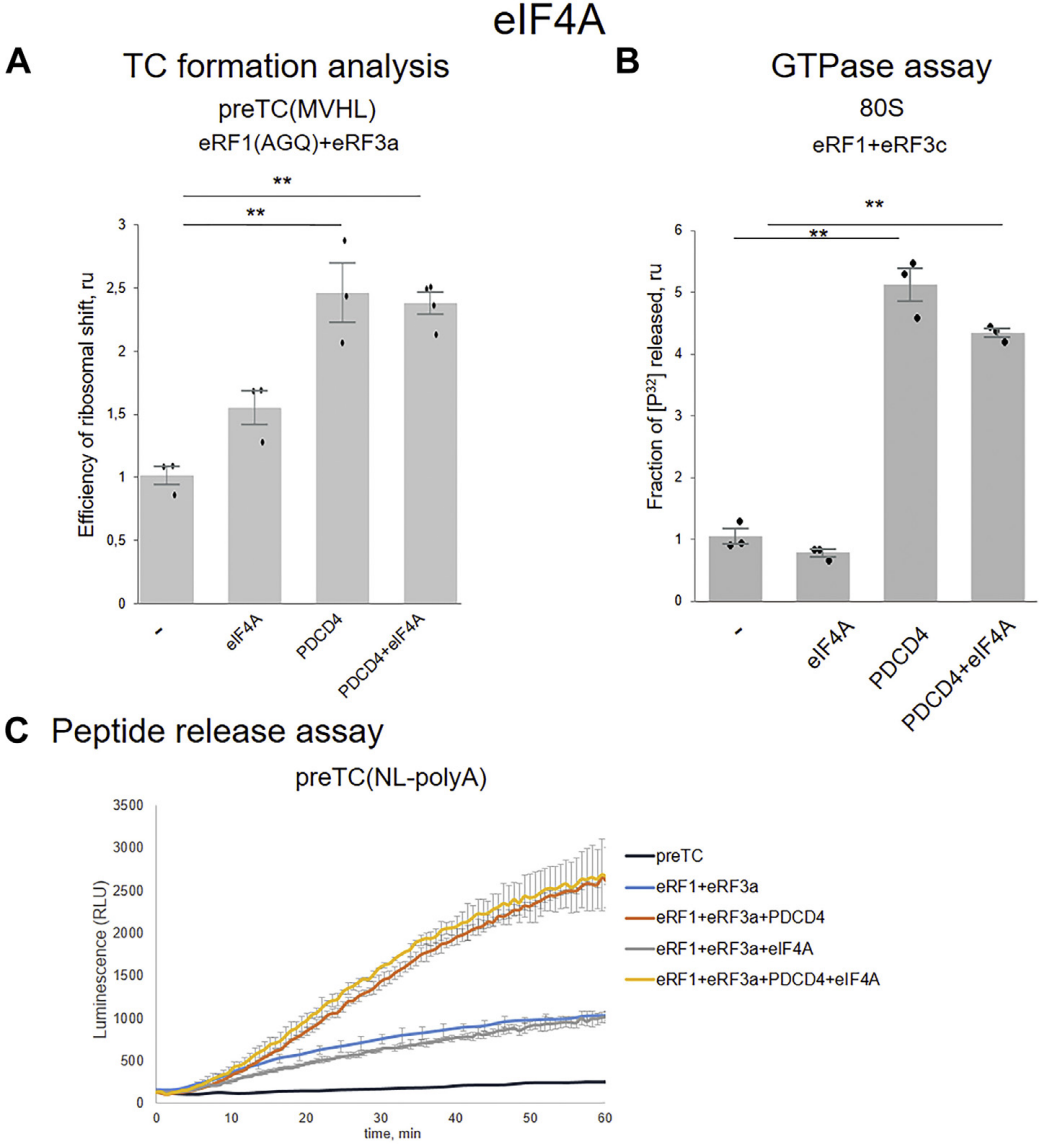
**Effect of eIF4A on PDCD4 activity in translation termination.***A*, TC formation analysis eRF1(AGQ)+eRF3a in the presence of eIF4A and PDCD4. The *black dots* show the replicate values. *B*, GTPase activity of eRF3c in presence eIF4A and PDCD4 (n = 3). The background was subtracted and each mean value was normalized to the value of eRF1(AGQ)+eRF3a or eRF1+eRF3c activity. The error bars represent the standard errors, *stars* (∗∗) mark a significant difference from the respective control *p* < 0.01, *t* test. *C*, effect of eIF4A on PDCD4-mediated peptide release in the presence of eRF1 and eRF3a (n = 3); time progress curves showing luminescence (in relative luminescence units, RLU) with NLuc luciferase released from ribosome complex upon treatment with the proteins of interest. The error bars represent the standard mean deviation.

This suggests that eIF4A does not affect translation termination. To test this more directly, we analyzed the efficiency of TC formation, hydrolysis of peptidyl-tRNA, and GTPase activity of eRF3a in the presence of both PDCD4 and eIF4A. We found that eIF4A did not affect TC formation (Fig. 6*A*), although it slightly reduced the GTPase activity of eRF3c (Fig. 6*B*). In addition, eIF4A did not alter the stimulation of peptidyl-tRNA hydrolysis by PDCD4 (Fig. 6*C*). Therefore, we conclude that the activities of PDCD4 in translation initiation *via* binding with eIF4A and in translation termination are independent processes.

### PABP-PDCD4 complex does not contain eRF3

After we revealed the stimulation of translation termination by PDCD4, we investigated the mechanism of this process. First, we sought to determine which component(s) of the TC interacted with PDCD4. We first verified the ability of recombinant PDCD4 to bind PABP (25, 44) using a pull-down assay. GST-tagged PDCD4 was incubated with PABP in solution and then immobilized on glutathione-sepharose. After cleavage of PDCD4 from GST by TEV protease, thereby eluted proteins were analyzed *via* western blot hybridization with antibodies against PDCD4 and PABP. We showed that PDCD4 expressed in *E.coli* efficiently bound to PABP in solution (Fig. S5*A*). We then analyzed the binding of PDCD4 to PABP and also to the release factors eRF1 and eRF3a-GTP (Fig. S5*A*). Upon coincubation with these proteins, only PABP was found in the complex with PDCD4.

Therefore, GST-PDCD4 can form a complex with PABP in solution and cannot interact with eRF3a and eRF1 under the tested conditions. Interestingly, we did not detect eRF3a and eRF1 in the complex with PABP and PDCD4, although PABP binds to PDCD4 through its N-terminal domain, and eRF3a through its C-terminal domain; and these interactions do not overlap spatially (Fig. 1*A*). We suggest that PDCD4 displaces eRF3 from the complex with PABP or cannot interact with PABP, which is associated with eRF3. This, in turn, can influence the activity of eRF3 in translation termination, because PABP loads eRF3a to the ribosome, which increases peptide release (26).

### PDCD4 binds to the ribosomal complexes

Next, we examined the ability of PDCD4 to interact with ribosomal complexes and subunits (Fig. S5*B*). For this purpose, we incubated PDCD4 with purified 80S or 40S subunits, fixed the complexes with 1% formaldehyde, and subjected them to a 10 to 30% (w/w) linear sucrose density gradient (SDG), resulting in the separation of unbound proteins from the ribosomes. Ribosome-associated proteins were probed with antibodies raised against ribosomal proteins RPS15, RPL9, and PDCD4. We observed that PDCD4 can form complexes with isolated 40S subunit and 40S in the 80S ribosome as it localized in the fractions of the gradient corresponding to the 40S ribosomal subunits. The results of the binding of PDCD4 to 40S were consistent with those previously obtained from interactions between PDCD4 and RSP13 (20)

**Figure 7:**
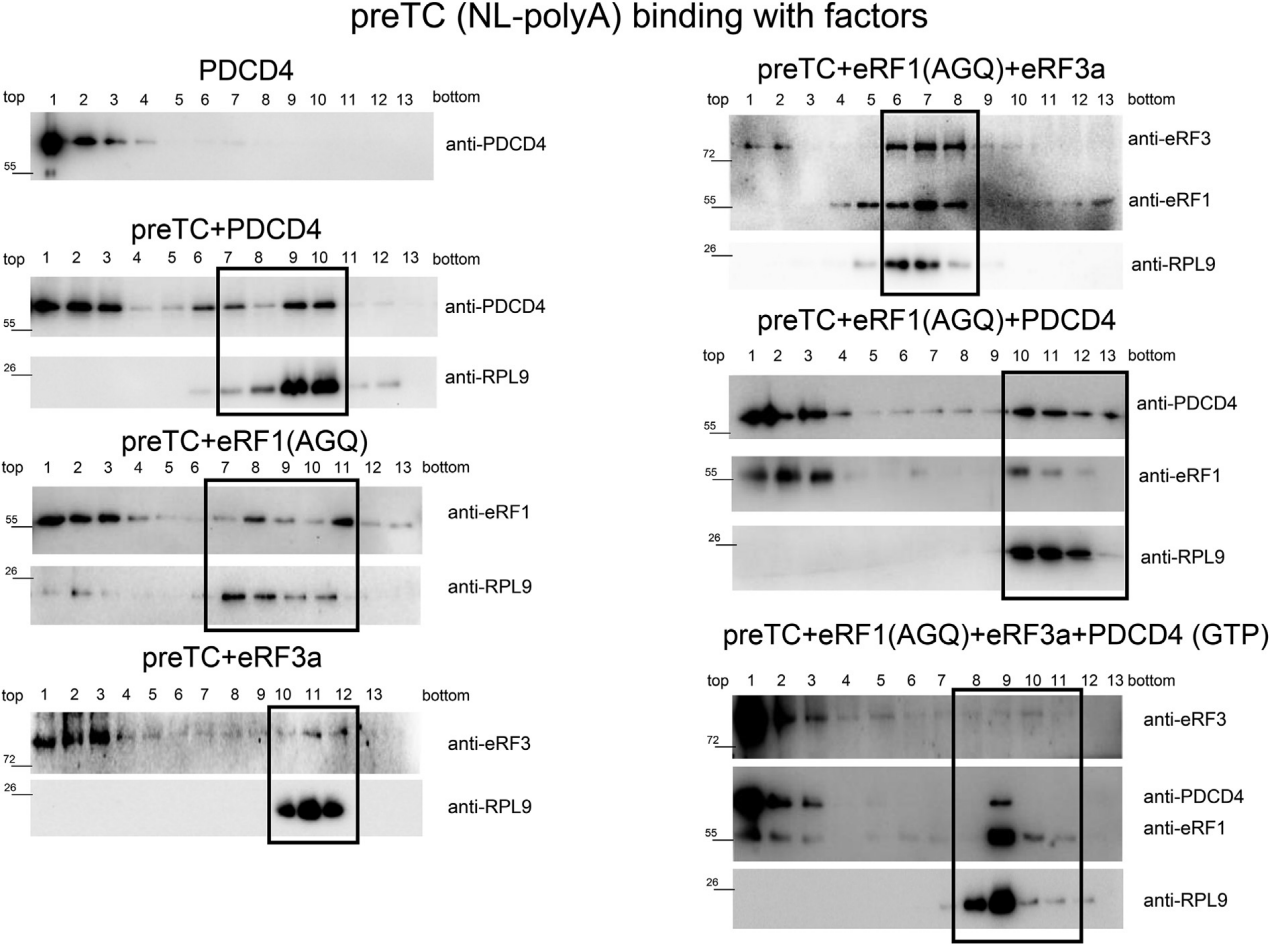
**PreTC NL binding with PDCD4 and the release factors.** Western blot analysis of SDG fractions. preTC NL binding with PDCD4, eRF1(AGQ), and eRF3a; the fractions of sucrose gradient corresponding to ribosome complexes are highlighted in *black*.

**Figure 8:**
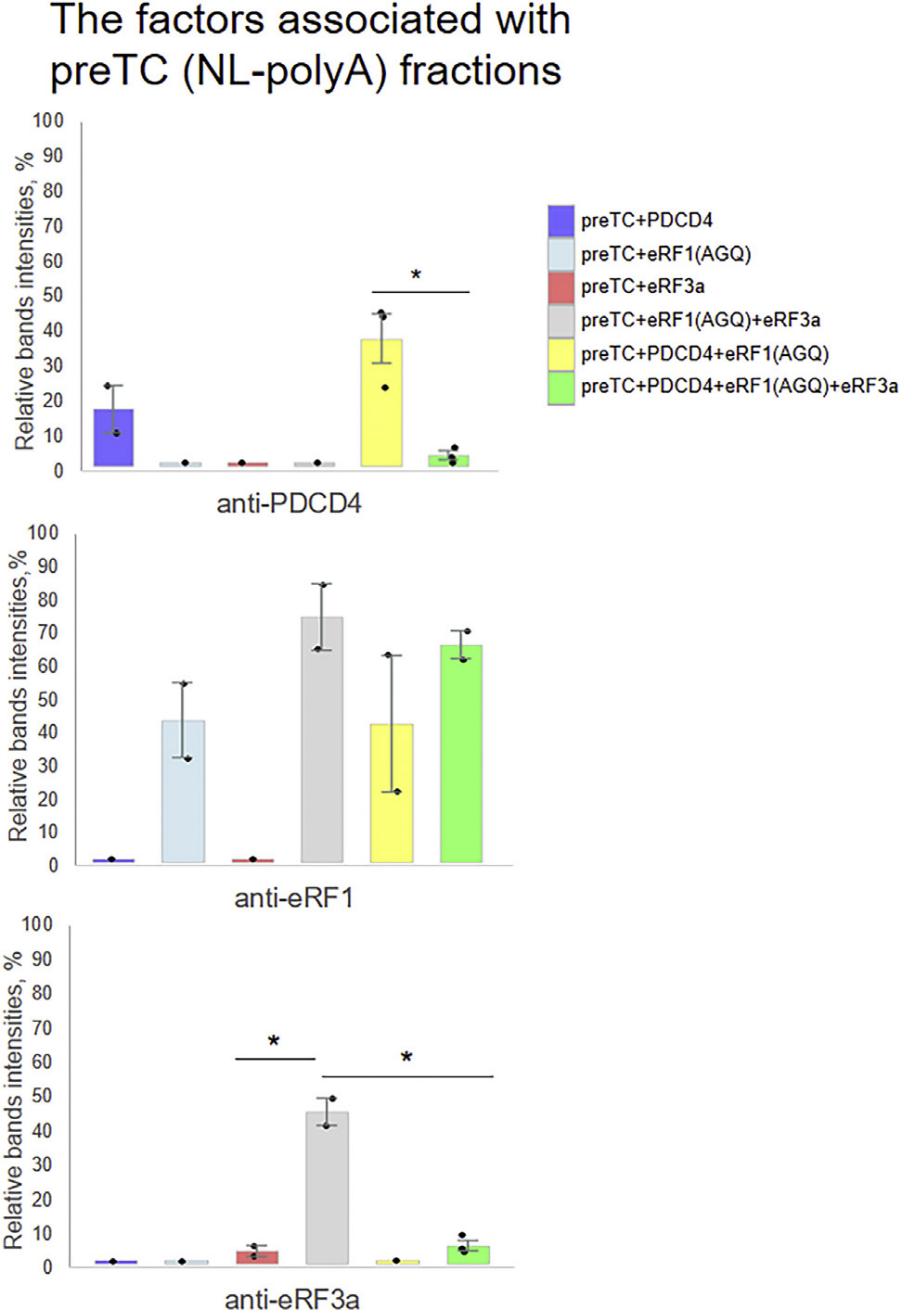
**Protein band distribution in ribosomal fractions depending on the added components.** Antibodies raised against PDCD4, eRF1, eRF3a, and RPL9 were used for detection. The *black dots* show the replicate values. The error bars represent the standard errors; *stars* (∗∗) mark a significant difference from the respective control *p* < 0.01, *t* test.

To study the ability of PDCD4 to interact with preTC or TC, we incubated PDCD4 with purified preTC(NL-polyA) in the absence or presence of release factors eRF1 and eRF3a-GTP, fixed the complexes with 1% formaldehyde, and subjected them to a 10 to 30% (w/w) linear SDG. Proteins bound to the preTC or TC were analyzed *via* western blot hybridization with antibodies raised against RPL9, eRF1, eRF3a, and PDCD4 (Fig. 7). We revealed that PDCD4 can form complex with the empty preTC (Figs. 7 and 8). Moreover, the amount of PDCD4, bound with the ribosomal complex, increased in the presence of eRF1 but dramatically decreased in the presence of both eRF1 and eRF3 (Fig. 8). Similarly, the amount of eRF3a in the TC increased in the presence of eRF1, but decreased after the addition of PDCD4 (Fig. 8). However, binding of eRF1 to the TC is independent of the presence of PDCD4 and increased in the presence of eRF3. In the control experiments, we did not observe the formation of oligomers of PDCD4 and release factors, which could migrate in the fractions of ribosomal complexes in the sucrose gradient (Fig. S5*C*).

Therefore, we found that PDCD4 forms complexes with preTC and TC in the presence of eRF1, bypassing the interaction with PABP (Figs. 7 and 8). Such interactions have not been detected previously. We conclude that PDCD4 is directly involved in translation termination through interactions with PABP and preTC, and can regulate the binding of eRF3 and PABP to each other and to the ribosomal complexes.

## Discussion

The role of PDCD4 in the initiation and elongation of eukaryotic translation has previously been reported (4, 5, 24). In the present study, we demonstrated the direct involvement of PDCD4 in translation termination. We showed that PDCD4 can bind to preTC and TC. We also demonstrated the PDCD4 activities at different stages of termination and identify the domains of the studied proteins that are important for this function.

We suppose that PABP, when not associated with release factors, is involved in the activity of PDCD4. We determined that the effect of PDCD4 on translation termination in the presence of PABP and poly(A) tail was increased (Figs. 1*B* and 2*B*). Therefore, although PDCD4 directly participates in translation termination, we propose that PABP can facilitate loading of PDCD4 to the ribosome during translation termination.

However, we believe that the pathways of enhancing translation termination by PABP alone and by PABP in the complex with PDCD4 are mutually exclusive, since eRF3 works in opposite ways. Using pull-down experiments, we did not observe any interactions between eRFs and PDCD4 (Fig. S5*A*). In a previous study, we demonstrated strong binding of PABP to eRF3a or to the eRF3a-eRF1 complex (26). Additionally, we showed that PDCD4 did not bind to the complex PABP-eRFs (Fig. S5*A*). Quantitative analysis of PDCD4 binding to the TC (Figs. 7 and 8) demonstrated the presence of a stable complex only in the absence of eRF3. In contrast, PABP interacts with preTC and TC in the complex with eRF3a (26).

PDCD4 interacts *via* its MA3m and MA3c domains with the eIF4A helicase (44), suppressing translation at the initiation stage. We showed that PDCD4 mutants were unable to interact with eIF4A and did not affect translation termination (Fig. 5*E*). Indeed, eIF4A did not influence the overall activity of PDCD4 upon termination stimulation. Therefore, we propose that eIF4A is not involved in the multicomponent termination complex.

**Figure 9:**
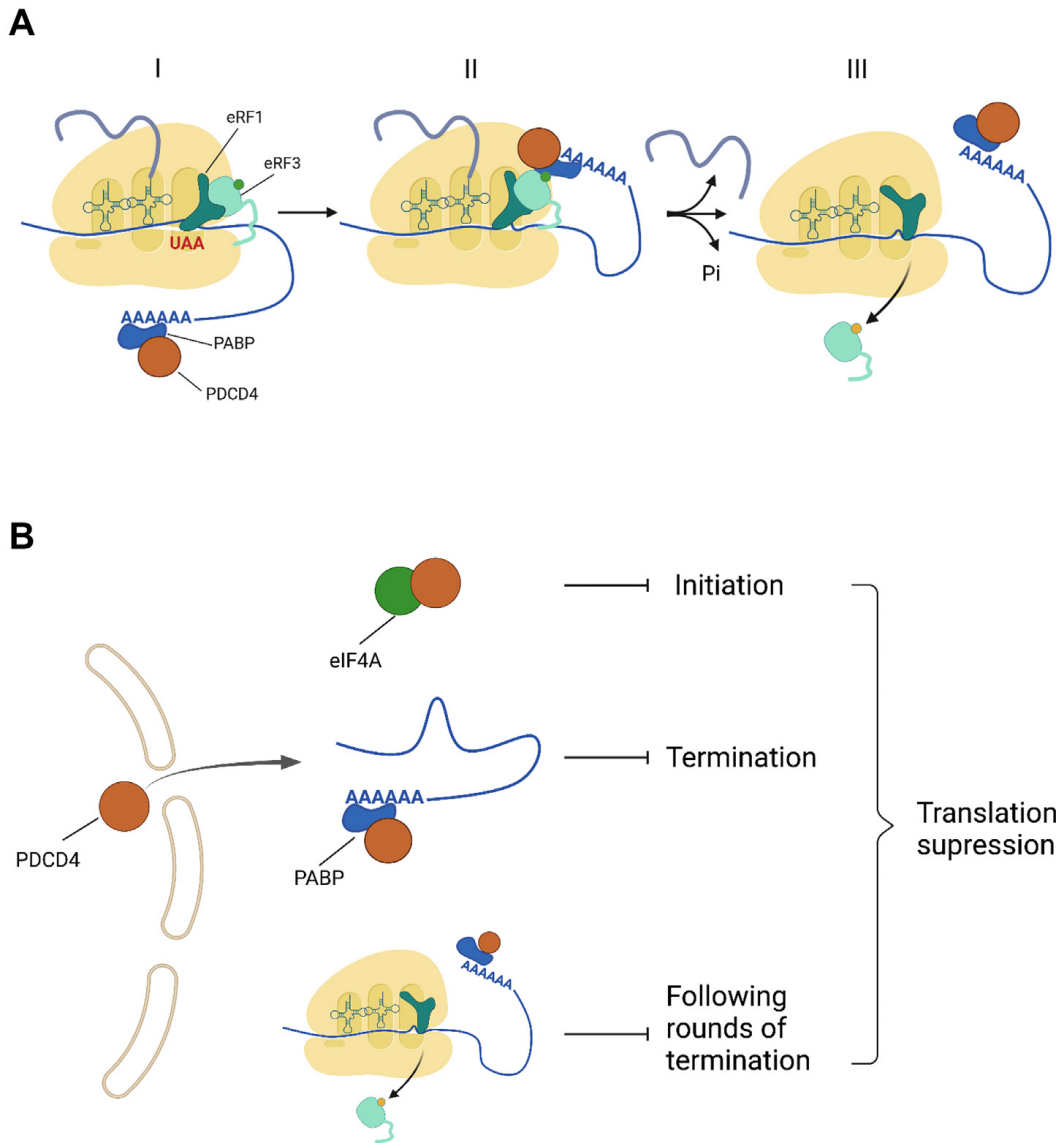
**Model for PDCD4 activity in translation.***A*, translation termination in the presence of PDCD4. (I) PDCD4 forms a complex with PABP at the poly(A) tail of mRNA; (II) complex of PDCD4-PABP binds to the TC bound with eRF1-eRF3-GTP and stimulates GTPase activity of eRF3; (III) after GTP hydrolysis and peptide release PDCD4-PABP and eRF3-GDP dissociate from the TC. *B*, suppression of translation by PDCD4. After stress signal PDCD4 translocates from the nucleus to the cytoplasm and binds to eIF4A, which suppresses initiation. PDCD4 binds to PABP and prevents formation of the PABP-eRF3 complex. This suppresses binding of release factors to the ribosome. PDCD4 stimulates termination on the already formed TC and displaces eRF3-GDP from the postTC. This prevents subsequent rounds of termination.

Overall, we propose the following mechanism of translation termination activation by PDCD4 (Fig. 9*A*). (I) PDCD4, either freely floating in the cytoplasm or in a complex with PABP and poly(A) tail, interacts with preTC and TC. It is possible that PABP stimulates PDCD4 loading onto the ribosome. In this step, PDCD4 also prevents the binding of eRF3 to PABP and disrupts the loading of new complexes of release factors to the ribosome by PABP. (II) In the second step, PDCD4 stimulates the GTPase activity of eRF3, and GTP hydrolysis triggers a conformational change in eRF1 and its positioning in the catalytic center of the ribosome. (III) In turn, it induces peptide release and dissociation of both PDCD4 and eRF3-GDP from the posttermination complex.

Previously, it was shown that PDCD4 is normally localized in the nucleus and migrates into the cytoplasm upon stress or apoptosis (45). Additionally PDCD4 suppresses cell translation at the initiation stage by binding to the initiation factor eIF4A, probably to prepare for cell stress conditions by transferring components into the stress granule (46). However, the entire process of translation cannot be stopped only by suppressing initiation, because those mRNAs that have already passed the initiation stage continue to be translated at the stages of elongation and termination and, in some cases, form closed-loop mRNA structures that are able to perform translation without new rounds of initiation (47). To disrupt the translation of such mRNAs, it would be reasonable to stimulate termination at stop codons and remove the release factors and 40S ribosome subunits from the pool of active components. We assumed that PDCD4 is involved in this process (Fig. 9*B*). First, it disrupts the binding of eRF3 to PABP, preventing the loading of release factors to the ribosomal A site and termination. Second, during stimulation of termination of the translating mRNA, PDCD4 displaces eRF3-GDP from the ribosome and PABP. This process prevents subsequent rounds of termination and transfers ribosomes to be inactivated in the stress granules. The presence of PDCD4, release factors, and 40S in the stress granules has been shown previously (48, 49, 50). Therefore, we believe that the biological significance of translation termination stimulation by PDCD4 is to transfer the components of protein synthesis to an inactive state.

We have shown for the first time that PDCD4 can participate in translation termination by stimulating the binding of release factors to the ribosome, increasing the GTPase activity of eRF3, and dissociating eRF3 from the posttermination complex. PDCD4 is a multifunctional regulator in the cell and is involved in many vital processes, such as the cell cycle (1), transcription (2, 3), translation initiation, and elongation (4, 24). Its overexpression inhibits cell invasion in many types of cancer (8, 9, 10, 51, 52). PDCD4 may play a role in differentiation and diseases such as diabetes and inflammation (6, 17). The described mechanism of PDCD4 activity in translation termination can be used in the development of therapeutic approaches for the treatment of cancer and other serious diseases in which PDCD4 participates.

## Experimental procedures

### Cloning and purification of PDCD4 and its mutant forms

cDNA encoding human PDCD4 (isoform 1, UniProt identifier: Q53EL6-1) and its domains MA3m 163 to 284 aa, MA3c 326 to 469 aa, and MA3m-c 163 to 469 aa were cloned into the pGEX-6p-1 vector (GE Healthcare) between *BamHI*/*XhoI* sites. For PDCD4 cloning, TATATCATATGGATGTAGAAAAT and TTTTCTCGAGGTAGCTCTCTGGTT primers were used. For the PDCD4 MA3m domain cloning, GGATCCGAAACTGTAGTTTTGCC and CTCGAGTCAACCTCCAGAGCCCCA primers were used. For PDCD4 MA3c domain cloning, GGATCCGGTGGGCAGCAATCTG and CTCGAGTCATGAAGGACAAAGATCTCTGAG primers were used. PDCD4 mutants E52A, K58E, K58A, D418A, and D414A were obtained based on the PDCD4 construct in the pGEX-6p-1 vector using the QuikChange Site-Directed Mutagenesis kit (Agilent) according to the manufacturer's recommendations. The resulting GST-tagged proteins were expressed in *E. coli* BL21 cells (Novagen) after induction with 1 mM isopropyl β-D-1-thiogalactopyranoside at 20 °C overnight, followed by purification using a glutathione-sepharose 4B beads (GE Healthcare) and elution with either TEV protease (kindly provided by Christiane Schaffitzel), which cleaves the GST-tag, or 10 mM glutathione in a buffer containing 50 mM Tris-HCl (pH 7.0), 100 mM KCl, 10 mM DTT, and 5% glycerol. Eluted proteins with or without GST-tag were then applied to a HiTrapQ column (GE Healthcare) and purified with a KCl gradient of 100 to 500 mM.

### Termi-Luc peptide release assay

A peptide release with NLuc was performed as previously described (34) with modifications. The assay allows measurement of NLuc release from preTCs assembled on NLuc mRNA in RRL. NLuc folds into the catalytically active form only after its release from the ribosome, which leads to luminescence in the presence of the substrate.

The NLuc mRNA was transcribed *in vitro* (T7 RiboMAX Express Large Scale RNA Production System, Promega) from a template containing the β-globin 5′-UTR, NLuc CDS, 3′-UTR derived from pNL1.1[Nluc] vector (Promega), and 50 nt poly(A) tail.

A reaction mixture containing 50% RRL (Green Hectares) was supplemented with 30 mM Hepes-KOH (pH 7.5), 50 mM KOAc, 1 mM Mg(OAc)_2_, 0.2 mM ATP and GTP, 0.04 mM 20 amino acids (Promega), 2 mM DTT, and 8 mM creatine phosphate to a final volume of 1 ml. The mixture was preincubated with 1 μM eRF1(AGQ) at 30 °C for 10 min, followed by the addition of NLuc mRNA to a final concentration of 8 μg/ml, resulting in the formation of preTCs. Next, KOAc concentration was adjusted to 300 mM, and the mixture was layered on a 10 to 35% linear sucrose gradient in buffer containing 50 mM HEPES-KOH, pH 7.5, 7.5 mM Mg(OAc)_2_, 300 mM KOAc, and 2 mM DTT. The gradients were centrifuged in a SW-41 Ti (Beckman Coulter) rotor at 18,000 rpm for 14 h. Fractions enriched with preTC NL were collected. PreTC NL was aliquoted, flash-frozen in liquid nitrogen, and stored at −80 °C. Peptide release was performed in a solution containing 7.5 nM preTC, 50 mM Hepes-KOH, pH 7.5, 0.25 mM spermidine, 2 mM DTT, 0.2 mM GTP, and 1% NLuc substrate (Nano-Glo, Promega) in the presence of release factors (8 nM eRF1(AGQ) alone or with 8 nM eRF3a/eRF3c) and proteins of interest (0.2 μM PABP, eIF4A, and PDCD4 or its MA3 domains). Luminescence was measured at 30 °C using a Tecan Infinite 200Pro (Tecan). The peptide release kinetic curves were generated, and the standard deviation was calculated using Microsoft Excel.

### Pretermination complex assembly

The 40S and 60S ribosomal subunits, as well as eukaryotic translation factors eIF2, eIF3, eEF1H, and eEF2, were purified from RRL or HeLa lysate, as previously described (29). The human translation factors eIF1, eIF1A, eIF4A, eIF4B, ΔeIF4G, ΔeIF5B, eIF5, PABP, and eRF1 were produced as recombinant proteins in *E. coli* strain BL21 with subsequent protein purification on Ni-NTA agarose and ion-exchange chromatography (29). Human eRF3a was expressed in insect Sf21 cells and purified by affinity chromatography using a HisTrap HP column (GE Healthcare) followed by anion-exchange chromatography using a MonoQ column (GE Healthcare) (26). mRNAs (with or without poly(A) tail) were transcribed *in vitro* (T7 RiboMAX Express Large Scale RNA Production System, Promega) from the linear fragment of the pET28-MVHL-UAA plasmid containing the T7 promoter, four CAA repeats, β-globin 5′-UTR, and ORF (encoding for the peptide MVHL), followed by the stop codon UAA and 3′-UTR, comprising the remaining natural β-globin coding sequence and, optionally, a 50 nt poly(A) tail. PreTCs on MVHL mRNA with and without poly(A) were assembled and purified as previously described (29, 39, 53). Briefly, ribosomal complexes were assembled in a 500 μl solution containing 37 pmol of mRNA. They were incubated for 15 min in buffer A (20 mM Tris acetate (pH 7.5), 100 mM KAc, 2.5 mM MgCl_2_, 2 mM DTT) supplemented with 400 U RiboLock RNase inhibitor (Thermo Fisher Scientific), 1 mM ATP, 0.25 mM spermidine, 0.2 mM GTP, 75 μg calf liver total tRNA (acylated with all or individual amino acids; Sigma Aldrich), 75 pmol human 40S and 60S purified ribosomal subunits, 200 pmol eIF2, 60 pmol eIF3, 400 pmol eIF4A, 150 pmol eIF4B, and 125 pmol each of human eIF2, ΔeIF4G, eIF1, eIF1A, eIF5, and ΔeIF5B for the formation of the 80S initiation complex. PABP (37 pmol) was added to the reaction to obtain the MVHL-poly(A)-PABP complex. After 15 min, 200 pmol of human eEF1H and 38 pmol of eEF2 were added to form the elongation complex. The ribosomal complexes were then purified *via* centrifugation in a Beckman SW55 rotor for 95 min at 4 °C and 48,000 rpm in a 10 to 30% (w/w) linear SDG prepared in buffer A with 5 mM MgCl_2_. Fractions corresponding to the ribosomal complexes were diluted threefold with buffer A containing 1.25 mM MgCl_2_ (to a final concentration of 2.5 mM Mg^2+^) and used in experiments.

### Termination complex formation efficiency assay

First, 0.03 pmol preTCs incubated with release factors (0.35 pmol eRF1(AGQ) alone or 0.02 pmol eRF1(AGQ)/eRF1 with 0.06 pmol eRF3a/eRF3c) and proteins of interest (1.5 pmol of PABP, 4 pmol of eIF4A, PDCD4, or its MA3 domains) at 37 °C for 10 min in the presence of 0.2 mM GTP or GDPCP were supplemented with equimolar amounts of MgCl_2_. The samples were analyzed using a primer extension protocol. The toe-printing analysis was performed with AMV reverse transcriptase and 5′-carboxyfluorescein-labelled primers complementary to the 3′-UTR sequences, as described previously (39). Using standard GeneScan conditions on an ABI Prism Genetic Analyser 3100 (Applera), cDNAs were separated *via* electrophoresis. The ribosomal shift efficiency was calculated as the proportion of cDNA corresponding to TCs using the formula TC/(TC+preTC). All data were normalized to the ribosomal shift efficiency calculated in the control experiments.

### GTPase assay

The ribosomal 40S and 60S subunits (1 pmol each) were associated at 37 °C for 10 min, and then, a GTPase buffer (10 mМ pH 8.0 Tris-HCl, 12 mM NH_4_Cl, 30 mM KCl, 6 mM MgCl_2_) containing 2 μM [γ-^32^P] GTP with a specific activity of 2.16 μC_i_/pmol was added. Next, 4 pmol of PDCD4, and its domains MA3m, MA3c, MA3m-c, or eIF4A were added to the reaction. The reaction was initiated by adding 1 pmol of eRF3a or eRF3c, and the final reaction volume was 10 μl. After incubation for 10 min at 37 °C, the reaction was stopped by adding 500 μl of a solution containing 5% charcoal in 50 mM NaH_2_PO_4_ to bind free GTP. Finally, the charcoal was precipitated *via* centrifugation at 12,000*g*, and 380 μl of the supernatant was subjected to liquid scintillation counting to quantify the released [^32^P] phosphate.

### Glutathione S-transferase (GST) pull-down assay

To study protein–protein interactions, *in vitro* pull-down assays were performed using purified proteins, as described previously with modifications (38). GST-PDCD4 (40 pmol) was incubated with 40 pmol of protein of interest (PABP, eRF1, eRF3a) in binding buffer (25 mM Tris-HCl (pH 7.5), 10% (v/v) glycerol, 100 mM KCl, 2.5 mM MgCl_2_, 2 mM DTT, 0.25 mM spermidine) containing 0.2 mM GTP equilibrated with MgCl_2_ at a final volume of 20 μl. The reaction mixture was incubated for 1 h at 4 °C, followed by the addition of 20 μl of 50% glutathione-sepharose 4B (GE Healthcare), equilibrated with the binding buffer for 5 min at 37 °C with shaking. After centrifugation (500*g*, 3 min), the supernatant was removed, and the resin was washed four times with 500 μl of binding buffer. Then, 1 μg of TEV protease diluted in 16 μl of binding buffer without GTP was added to the resin and incubated for 10 min at 37 °C. The linker between GST and PDCD4 in the GST-PDCD4 molecule was cleaved, releasing the complex of PDCD4 and its interacting proteins into the solution. The supernatant was collected through a mini-spin column (500*g*, 2 min), leaving the resin on the filter. Then, the supernatant was diluted to 30 μl with sample buffer. Ten microliters of the final sample was used for Western blot analysis using the following antibodies: anti-PDCD4 (Bethyl, A301-107A) anti-eRF3 (Cell Signaling Technology, 14980S), anti-eRF1 (Abcam, 153731), and anti-PABP (Abcam, 21060). As positive control for antibodies, inputted proteins were added in the following dilutions relative to the experimental samples: ×1/5 for GST-PDCD4, ×1/600 for eRF1, eRF3, and PABP.

### Ribosome and ribosomal complex binding assay

1.35 pmol purified preTCs NL or 10 pmol of 40S and 80S ribosome were incubated with 10 pmol eRF3a, 10 pmol eRF1(AGQ), and 10 pmol PDCD4 either separately or together in buffer containing 20 mM Tris-HCl pH 7.0, 10 mM MgCl_2_, 2 mM DTT, 0.1 mM spermidine with 0.2 mM GTP equilibrated with 0.2 mM MgCl_2_ at 37 °C for 10 min. The reaction volume was 100 μl. Subsequently, TCs were incubated with 1% formaldehyde at 4 °C for 1 h. Glycine was added up to 0.1 M to stop the cross-linking reaction. The preTC NL was centrifuged into a 10 to 30% (w/w) linear SDG, as described above. The whole gradient was fractionated into 13 equal fractions (385 μl), followed by precipitation with 10% trichloroacetic acid. The protein pellets were dried and analyzed using western blotting analysis with corresponding antibodies raised against PDCD4 (Bethyl Laboratories, A301-107A), eRF1 (Abcam, 153731), eRF3 (Cell Signaling Technology, 14980S), RLP9 (Abcam 182556), and RPS15 (Abcam, 154936).

### Statistical data processing

All experiments were performed with at least two replicates. A two-tailed *t* test was used to compare the mean values between the groups. For multiple comparisons, the Bonferroni correction was used.

## Data availability

The data that support the findings of this study are contained within the article and the supporting information. All source data generated for this study are available from the corresponding author (Dr Elena Alkalaeva; alkalaeva@eimb.ru) upon reasonable request.

## Supporting information

This article contains supporting information.

## Conflict of interest

The authors declare that they have no conflicts of interest regarding the contents of this article.

## Figures and Tables

**Supplemental Figure S5:**