# Supplementary material for: Discovery of a novel role of tumor suppressor PDCD4 in stimulation of translation termination
Source: J Biol Chem. 2021 Oct 1;297(5):101269. doi: 10.1016/j.jbc.2021.101269 (PMC8551656; doi:10.1016/j.jbc.2021.101269)
Supplement: Supporting information [file mmc1.docx]

# Supporting information

**S1 Figure.**Scheme of NL, MVHL and MVHL-poly(A) mRNA constructs.

**S2 Figure.** (A) Peptide release of NLuc luciferase upon treatment with different concentrations of eRF1 and eRF3a (8–64 nM) in the presence or absence of 400 nM PDCD4. (B) Multiple alignment of PDCD4 from different organisms (17–61 a.a., 393–452 a.a.), grey highlights show similar amino acids. The positions, mutated in the study, are indicated in red.

**S3 Figure.** (A) Raw data of TC formation analysis shown in Figure 2B. (B) Raw data of TC formation analysis shown in Figure 2C. The positions of the preTCs and TCs are labelled by white and black triangles, respectively. Red stars indicate samples where ribosomal shift is enhanced. (C) Effect of non-hydrolysable GTP analogues on peptide release in the presence of eRF1 and eRF3a (n=2), time progress curves showing luminescence (in relative luminescence units, RLU) with NL released from the ribosome complex upon treatment with the proteins of interest. Error bars represent standard deviation.

**S4 Figure.** (A) Raw data of TC formation analysis shown in Figure 3A. (B) Raw data of TC formation analysis shown in Figure 3C. (C) Raw data of TC formation analysis shown in Figure 4A. The positions of the preTCs and TCs are labelled by white and black triangles, respectively. Red stars indicate samples where ribosomal shift is enhanced.

**S5 Figure.** (A) In vitro pull-down assay of PABP/eRF1/eRF3a with PDCD4 after TEV-protease cleavage of GST-PDCD4. As positive controls for antibodies, inputted proteins were added in the following dilutions relative to the experimental samples: x1/5 for GST-PDCD4, x1/600 for eRF1, eRF3, and PABP. The experiment was performed qualitatively in two replicates. (B) Western blot analysis of SDG fractions of PDCD4, PDCD4+40S, PDCD4+80S using antibodies raised against PDCD4, RPL9, and RPS15. The profile of PDCD4 distribution in SDG was reused from Figure 7 as a representative control. (C) Negative controls for the preTC binding analysis. Distribution in the SDG of eRF1(AGQ) or eRF3a mixed with PDCD4. Western blot analysis of the SDG fractions. Antibodies raised against PDCD4, eRF1, and eRF3a were used for detection.
